# In-depth analysis of *Bacillus subtilis* proteome identifies new ORFs and traces the evolutionary history of modified proteins

**DOI:** 10.1038/s41598-018-35589-9

**Published:** 2018-11-22

**Authors:** Vaishnavi Ravikumar, Nicolas C. Nalpas, Viktoria Anselm, Karsten Krug, Maša Lenuzzi, Martin Sebastijan Šestak, Tomislav Domazet-Lošo, Ivan Mijakovic, Boris Macek

**Affiliations:** 1 Novo Nordisk Foundation Center for Biosustainability, Technical University of Denmark, Kongens Lyngby, Denmark; 2 Proteome Center Tuebingen, Interfaculty Institute for Cell Biology, University of Tuebingen, Tuebingen, Germany; 3 Laboratory of Evolutionary Genetics, Ruđer Bošković Institute, Bijenička cesta 54, HR-10000 Zagreb, Croatia; 4 Catholic University of Croatia, Ilica 242, HR-10000 Zagreb, Croatia; 5 Systems and Synthetic Biology, Department of Chemical and Biological Engineering, Chalmers University of Technology, Gothenburg, Sweden; 6 Present Address: Proteomics Platform, The Broad Institute of MIT and Harvard, Cambridge, MA USA

## Abstract

*Bacillus subtilis* is a sporulating Gram-positive bacterium widely used in basic research and biotechnology. Despite being one of the best-characterized bacterial model organism, recent proteomics studies identified only about 50% of its theoretical protein count. Here we combined several hundred MS measurements to obtain a comprehensive map of the proteome, phosphoproteome and acetylome of *B. subtilis* grown at 37 °C in minimal medium. We covered 75% of the theoretical proteome (3,159 proteins), detected 1,085 phosphorylation and 4,893 lysine acetylation sites and performed a systematic bioinformatic characterization of the obtained data. A subset of analyzed MS files allowed us to reconstruct a network of Hanks-type protein kinases, Ser/Thr/Tyr phosphatases and their substrates. We applied genomic phylostratigraphy to gauge the evolutionary age of *B. subtilis* protein classes and revealed that protein modifications were present on the oldest bacterial proteins. Finally, we performed a proteogenomic analysis by mapping all MS spectra onto a six-frame translation of *B. subtilis* genome and found evidence for 19 novel ORFs. We provide the most extensive overview of the proteome and post-translational modifications for *B. subtilis* to date, with insights into functional annotation and evolutionary aspects of the *B. subtilis* genome.

## Introduction

*Bacillus subtilis* is an aerobic, endospore forming, rod-shaped soil bacterium from the phylum Firmicutes and family Bacillaceae. It is universally regarded as a model organism for bacteria in general and Firmicutes in particular. Many natural phenomena, such as bacterial chromosome replication, sporulation, swarming, natural competence and carbon catabolite repression have been characterized in depth using *B. subtilis*, making it one of the best-characterized bacterial organisms to date. It is also widely used as a cell factory for production of industrial enzymes and chemicals^1–3^. Many clinically relevant bacterial pathogens, such as *Bacillus anthracis*, *Listeria monocytogenes* and *Staphylococcus aureus* are closely related to *B. subtilis*, making it a significant cellular system for research on new antimicrobials^4^.

Shotgun proteomics generates valuable information from large-scale analysis of protein expression, post-translational modifications (PTMs), and protein–protein interactions, e.g. in conjunction with immunoprecipitation or cross-linking. Several large-scale proteomics datasets of *B. subtilis* have been published. Some of the earlier studies employed two-dimensional protein gel electrophoresis in combination with N-terminal amino acid sequencing^5^ or MALDI-MS^6^. More recent studies employed shotgun proteomics that enables in-depth proteome coverage under various biological conditions, reaching identification of about 2,200 proteins in exponentially growing *B. subtilis* cells^7,8^, which represents 52% of the 4,197 proteins encoded in the *B. subtilis* genome.

In *B. subtilis*, Ser/Thr/Tyr protein phosphorylation has been shown to play key regulatory roles, involving cellular processes such as carbon catabolite regulation^9–11^, DNA replication, spore development^12,13^, or spore germination^14–16^. *B. subtilis* Ser/Thr- and Tyr-protein kinases have recently been shown to engage in inter-kinase cross-phosphorylation, suggesting that their signal transduction pathways may be connected or overlapping^17^. The largest phosphoproteome map of *B. subtilis* reported identification of 225 phosphorylation events^18^. Another abundant and reversible PTM, protein acetylation, is recognized to influence metabolic pathways in bacteria^19–21^. Apart from its involvement in metabolic reactions, the acetyltransferase AcuA has also been shown to play a key role in sporulation in *B. subtilis*^22^. Recent studies identified between 600–700 acetylated proteins in *B. subtilis*^23,24^.

Here we provide a comprehensive resource of the proteome, phosphoproteome and acetylome of *B. subtilis* subsp. *subtilis* str. 168 under various growth conditions, obtained by processing over 1,600 LC-MS/MS runs, previously acquired in our laboratory on the same technological platform. We use this dataset to reconstruct a network of Hanks-type protein kinases, Ser/Thr/Tyr phosphatases and their substrates, to correlate evolutionary age of proteins with their expression and PTMs using genomic phylostratigraphy and to re-annotate *B. subtilis* open reading frames (ORFs) by mapping acquired MS/MS spectra onto the genome sequence using proteogenomics^25^. Our resource provides the most extensive overview of proteome and PTM data for *B. subtilis* to date, with insights into functional and evolutionary aspects of the *B. subtilis* genome.

## Results

### Comprehensive Map of *B. subtilis* Proteome Covers 75% of Predicted ORFs

Mass spectra from 1,688 proteome, phosphoproteome and acetylome LC-MS/MS runs, acquired over several years on similar nano-LC-MS (Orbitrap) platforms, were processed together using MaxQuant software^26^. This resulted in the identification of 3,159 proteins at the false discovery rate of 1% (protein level), covering 75.26% of the theoretical *B. subtilis* proteome (Supplementary Data S1). The average protein sequence coverage of 58.8% and small number of proteins (46), detected by a single peptide, point to extensive sampling of the expressed *B. subtilis* proteome.

**Figure 1:**
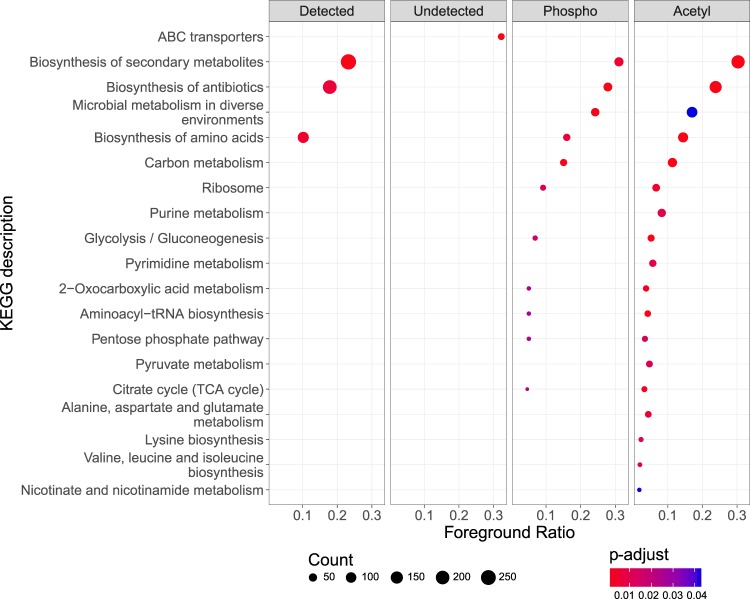
Functional annotation of *Bacillus subtilis* proteins. Proteome categories comprise: Detected = all identified proteins; Undetected = proteins not identified in our study; Phosphorylated = all proteins found phosphorylated at least once; Acetylated = all proteins found acetylated at least once. On the x-axis, the foreground ratio is plotted for each KEGG pathway; these ratios represent the number of proteins per category and per pathway divided by the total number of proteins per pathway. On the y-axis, the KEGG pathway description is displayed. Color gradient corresponds to the multiple correction testing adjusted *p*-value from lowest (red) to highest (blue). The size of the dots corresponds to protein count per KEGG pathway for each category.

Functional annotation^27,28^ of detected proteome, including phosphorylated and acetylated proteins, and its comparison to *B. subtilis* theoretical proteome revealed a consistent over-representation of KEGG pathways involved in biosynthesis of secondary metabolites, antibiotics and amino acids (adjusted *p*-value ≤ 0.05) (Fig. 1 and Supplementary Fig. S1). Despite of extensive peptide sequencing efforts, almost 25% of the theoretical proteome escaped detection in the current study (undetected proteome). Interestingly, in this part of the proteome, only the KEGG pathway associated with ABC transporters was over-represented (Fig. 1), while uncharacterized proteins were the most prominent group (Supplementary Fig. S1). In addition, over-representation analysis based on gene ontology (GO) cellular component revealed that GO terms associated with plasma membrane were enriched in the undetected proteome (Supplementary Fig. S1), pointing to lower accessibility of the membrane proteome to MS analysis.

### Protein Phosphorylation Predominantly Occurs On Proteins Involved In Metabolic Pathways

Analysis of the *B. subtilis* phosphoproteome (631 LC-MS/MS runs) resulted in identification of 1,085 phosphorylation events on 488 proteins, of which 866 were localized to a specific Ser/Thr/Tyr residue (localization probability ≥0.75) and 521 were identified with high confidence (i.e. posterior error probability [PEP] ≤ 0.001) (Supplementary Data S1). About 45% percent of identified phosphoproteins were detected with a single phosphorylation event. In agreement with previous studies, most of the localized phosphorylation events were observed on serine (65.1%), followed by threonine (18.7%) and tyrosine (16.2%) residues (Supplementary Fig. S2).

Most of the phosphorylated proteins were involved in biosynthesis of secondary metabolites (19.1%) and antibiotics (17.1%), followed by carbon metabolism (9.5%) and amino acid metabolism (9.8%) (Supplementary Fig. S1). We used motif-x^29,30^ to detect kinase amino acid sequence motifs within identified phosphorylated peptides that could correspond to kinase recognition features. While no significant motifs were found amongst Thr- and Tyr-phosphorylated peptides, analysis of serine phosphorylated peptides revealed five putative motif patterns, all of which were enriched in serine residues at various positions upstream or downstream of the phosphorylated residue (Supplementary Fig. S3).

### Kinase-Substrate Network Analysis Reveals Multiple Proteins Targeted By Sty Kinases And Phosphatases

**Figure 2:**
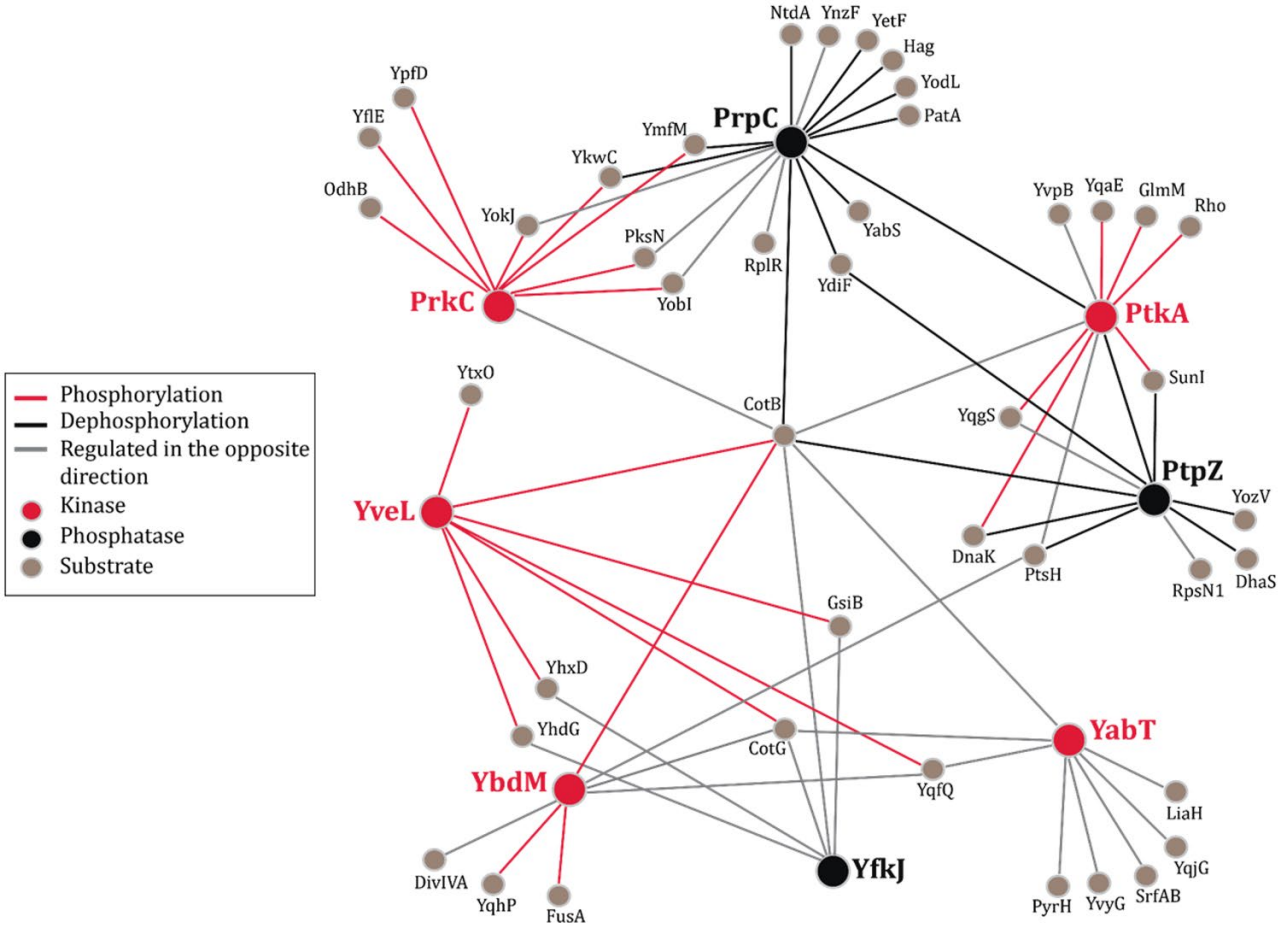
Interaction network of regulated putative substrates (direct or indirect) of all analyzed kinases and phosphatases. Kinases and their phosphorylation events are depicted in red color and phosphatases and their dephosphorylation events are depicted in black color. Proteins that are regulated by the respective kinases and phosphatases in the opposite direction are indicated by light grey lines. Respective putative direct substrates are depicted in brown. All proteins in the above interaction network are referred to as nodes and their interactions as edges.

A considerable fraction of our dataset (631 LC-MS/MS runs) were derived from quantitative SILAC^31^ MS studies comparing occupancy of phosphorylation sites in kinase or phosphatase knock-out strains to that of the wild type (see Methods and Supplementary Method S1). We reconstructed an interaction network of phosphorylation events that were up- or down-regulated in each of the analyzed kinase (*prkC, yabT, ybdM, ptkA, ptkB*) and phosphatase (*prpC, ptpZ, yfkJ*) knock-out strain (Fig. 2). As a threshold for regulated (changing) phosphorylation events we used a cutoff of +/−1.5 in log_2_ scale. Several well-known regulatory phosphorylation events were strongly regulated: Y225, Y227 and Y228 on the BY-kinase PtkA^32–34^; S46 on the histidine-containing phosphocarrier protein HPr^35^; S680 on the elongation factor G^36,37^; and S100 on the phosphoglucosamine mutase GlmM^38^. Among the remaining regulated events we detected several phosphorylation sites that have not been observed before, presenting promising leads for follow-up studies. The most striking novel feature revealed by this network analysis were spore coat proteins CotB and CotG that were found multiply phosphorylated on Ser/Thr residues. Phosphorylation events on S253 and S254 of CotB were consistently detected in all knock-out conditions, suggesting a synergistic or backup action of all Ser/Thr kinases and phosphatases. In addition, our network analysis highlighted multiple nodes potentially regulated by both, kinases and phosphatases, suggesting that they act in the same pathways and are likely to be tightly regulated (Fig. 2). For example, YkwC (beta-hydroxyacid dehydrogenase) and YmfM (a cell shape determination protein) are targets of the kinase PrkC and phosphatase PrpC^36^; DnaK (heat shock protein) and SunI (bacteriocin producer immunity protein) are regulated by the kinase PtkA and phosphatase PtpZ^34^.

### Global Acetylome Analysis Reveals Diverse Putative Functions of Lysine Acetylation

The acetylome analysis (19 LC-MS/MS runs) resulted in identification of 4,893 acetylation events on 1,277 proteins, the majority of which were localized to a specific lysine residue (localization probability ≥0.75) and were confidently identified (PEP ≤ 0.001) (Supplementary Data S1). Notably, 53% of detected proteins were either singly or doubly acetylated (Supplementary Fig. S2). Similar to the phosphoproteome, the functional annotation and over-representation of the identified acetylome revealed many proteins involved in secondary metabolite production and biosynthesis of antibiotics and amino acids (Fig. 1 and Supplementary Fig. S1). Proteins involved in sporulation (Spo0B, Spo0A, Spo0F, Spo0J, Spo0M, SpoIIAA, SpoVAD, SpoVR, SpoVS, KinE, KapB) were found acetylated on multiple lysine residues. Interestingly, several Rap proteins were also detected with multiple acetylation events. Rap proteins belong to the family of tetratricopeptide-containing regulatory proteins in *B. subtilis* and are involved in processes such as sporulation or competence development. Using motif-x, 16 sequence motif patterns were detected among all acetylated peptides, all of which contain either one or more positively (K) or negatively (E) charged amino acid residues (Supplementary Fig. S3).

### Genomic Phylostratigraphy Provides Insights Into Evolutionary Age Of *B. Subtilis* Proteins

**Figure 3:**
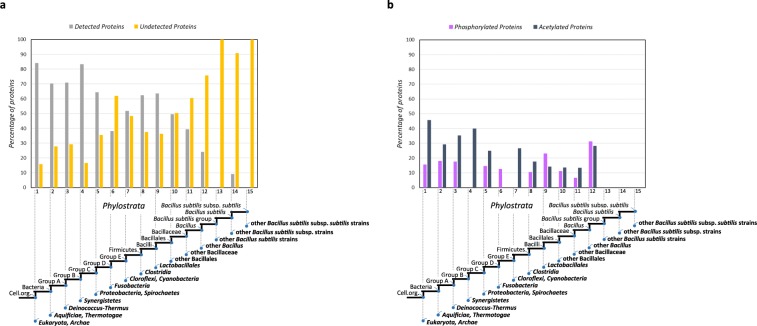
The phylostratigraphy map of *B. subtilis* subsp. *subtilis* str. 168 genome depicting distribution of: (**a**) detected and undetected, (**b**) phosphorylated and acetylated proteins. Estimated evolutionary origins of individual genes are mapped on the depicted reference evolutionary tree (x-axis). All *B. subtilis* genes have been distributed into 15 groups (phylostrata) according to the estimated point of emergence of their protein family founders. In panel a), the y-axis denotes the percentage of detected and undetected proteins, out of the total number of proteins sorted to each phylostratum. In panel b), the y-axis denotes the percentage of modified proteins out of the total number of proteins assigned to each phylostratum.

We next asked whether this comprehensive dataset may reveal information on the evolutionary history of the unmodified, phosphorylated and acetylated *B. subtilis* proteins. To this end, we constructed a reference phylogenetic tree for *B. subtilis*, and populated its nodes with *B. subtilis* genes originating at different evolutionary levels (see Methods). This genomic phylostratigraphy approach resulted in distribution of all *B. subtilis* genes in 15 phylostrata (ps), with ps1 being the oldest and ps15 being the most recent phylostratum (Fig. 3). It should be noted that the older phylostrata contained most of *B. subtilis* proteins, while more recent phylostrata were not as heavily populated. From the proteome perspective, the distribution of evolutionary ages of expressed (detected) versus non-expressed (undetected) proteins exhibited a clear trend (Fig. 3a): expressed proteins represented a dominant fraction (70–80%) in the oldest phylostrata (ps1-4), and their percentage then continually dropped, to reach zero in some of the most recent phylostrata (ps13 and ps15). This observation can be supported by the fact that essential proteins, usually traced to evolutionary older founder genes, tend to be more abundant and therefore more likely to be experimentally detected^39^. Conversely, younger phylostrata contained proteins with more specialized functions (such as specific ABC transporters), that may likely be detected only under specific growth conditions.

Age distribution of expressed (detected) *B. subtilis* proteins that undergo phosphorylation and acetylation is shown in Fig. 3b. Acetylation levels were the highest in ps1-4, reaching over 40% in some phylostrata. Phosphorylation levels were generally lower, in the range of 10–20%. The only exceptions were relatively recent ps9 and ps12, where over 20 and 30% of proteins are phosphorylated, respectively. Phosphorylated proteins of known function in ps9 and ps12 are involved in sporulation and induction of the prophage SP-β, respectively. Regulation of spore development is known to rely heavily on protein phosphorylation^13,15,40^, and it is therefore plausible that more recent components of the sporulation machinery would have a high propensity of being phosphorylated. Interestingly, sporulation and induction of the SP-β prophage are in fact co-regulated^41^. The remaining phosphorylated proteins from ps9 and ps12 were of unknown function. Proteins from ps13 and ps15 were not detected, hence no PTMs could be detected either. In addition, there were no PTMs detected on expressed proteins from ps14. Taken together, these results demonstrate the presence of PTMs even in the oldest phylostrata, pointing to the possibility that they were present and likely functional very early in protein evolution.

### Proteogenomics Identifies Novel Translated *B. Subtilis* ORFs Of Uncharacterized Function

**Figure 4:**
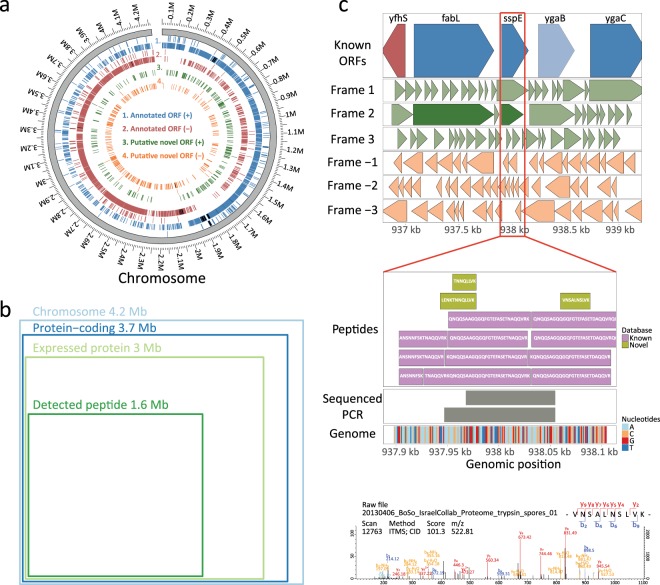
MS/MS quality and genomic coverage. (**a**) Circos graph representation of *B. subtilis* genome, including the annotated and potentially novel ORFs expressed in this study. (**b**) Venn diagram illustrating the MS coverage at peptide and protein levels in context of *B. subtilis* genome. (**c**) The genomic region visualization for seq_51322. Top panel includes known ORFs (in blue for + strand and in red for − strand) and all ORFs generated from six-frame genome translation (in green for + strand and in orange for − strand); color lightness corresponds to whether ORF was identified or not in our data (dark color for expressed ORF and light color for non-expressed ORF). The middle panel is zoomed around the expressed novel ORF of interest for visualization of peptide sequences (in khaki are peptides mapping to novel ORF and in purple are peptides from known ORF) and RT-PCR nucleotide sequences at this genomic location. The bottom panel contains the MS/MS spectra of the top scoring novel PSM.

Establishment of this large proteome dataset enabled us to address genome coverage and existence of yet undiscovered ORFs in *B. subtilis*. To this end, we re-processed MS data against a protein database generated from ORF translation in all six frames. This revealed 3,886,317 non-redundant peptide-spectrum matches (PSM) coming from the target database, 5,193 PSM unique to the six-frame database and 1,015 PSM from the decoy database (Supplementary Data S2). Distribution of expressed and annotated ORFs (Fig. 4a) confirmed the previously observed co-orientation of replication and transcription in bacterial genomes^42^. Detected peptides mapped to 1.6 Mb of the *B. subtilis* genome, corresponding to 37.8% of the complete chromosome (Fig. 4b). Each detected nucleotide was covered on average by 98.4 MS/MS spectra, whereas the median coverage was 10x (Supplementary Fig. S4).

Initially, a total of 631 unique peptide sequences were identified, corresponding to 532 potentially novel ORFs. Following a stringent PEP filtering step, the number of potential novel ORFs was reduced to 90. To stratify these novel ORFs, we integrated results from protein BLAST analyses, Levenshtein distance and nucleotide distance between neighboring ORFs. We then focused on 19 novel ORFs detected with two or more unique peptides. Out of these, six ORFs had alternate start regions, two had erroneous terminations, four were known in other bacterial species, five contained amino acid variations and two were uncharacterized (Supplementary Fig. S4).

For validation by RT-PCR and Sanger sequencing, we selected seven novel ORFs, of which four passed our stringent filtering and three did not (Supplementary Data S3). We confirmed the presence of transcribed mRNA for all four post-filtering novel ORFs (Table 1), while the three pre-filtering novel ORFs could not be validated due to the absence of RT-PCR products. Figure 4c shows the genomic region for one of the confirmed, uncharacterized ORFs together with associated novel peptides (ORF ID = seq_51322). Notably, seq_51322 ORF is located on the +2 frame and overlaps a known ORF (*sspE*, UniProt ID = P07784) on the +1 frame. The sequence of seq_51322 ORF did not align with SspE. The same visualization strategy was performed for other uncharacterized ORFs (Supplementary Fig. S5).

**Table 1:** Novel ORFs selected for RT-PCR validation.

Database ID	Sequences	PCR validated	ORF length (aa)	Annotation
seq_154909	FIKISRSESASK, ISRSESASK	Yes	70	Uncharacterised (no BLAST results)
seq_51322	LENKTNNQLLVK, TNNQLLVK, VNSALNSLVK	Yes	69	Uncharacterised (no BLAST results)
seq_163507	NVMYRLCYFLSEK, SPGMFSGLFVFK	No	57	Unclear/false positive
seq_134853	AFGRMLRLILMMPMK, RWLALSSRQSCCLIGNTIIGAWISSSNEFIN	No	51	Unclear/false positive
seq_145510	SVMLSAVQELLCGSILK, TLLNYFLRPAMNLFPAK	Yes	45	Erroneous termination of P42977
seq_49263	SLRYLHQETVQTSK, YLHQETVQTSKPSSR	Yes	20	Erroneous termination of P12043
seq_223100	MNISSNVCRPMIMLK, NISSNVCRPMIMLK	No	17	Unclear/false positive

Potentially novel ORFs identified in this study, including associated peptide sequences and possible annotation for these events.

## Discussion

In the current study we identified 75.26% of *B. subtilis* theoretical proteome, making this the most comprehensive *B. subtilis* proteome dataset reported to date. Comparison of the results with other published large-scale proteomics datasets^7,8^ showed that 1,748 proteins (41.64%) were observed across all studies (Supplementary Fig. S6). Combined, these studies detected 3,324 *B. subtilis* proteins, accounting for 79.2% of its theoretical proteome. Remarkably, more than 20% of the *B. subtilis* proteome was not detected by shotgun proteomics, most likely due to the use of minimal media and defined laboratory conditions in corresponding studies. Conversely, most of the *B. subtilis* gene products have been detected at the transcript level^43^ and most proteins detected in our study have been reported to be transcribed (Supplementary Fig. S6). Comparison of the identified phosphorylated and acetylated proteins to the largest published phosphoproteome and acetylome datasets of *B. subtilis*^18,23,24^ revealed that a majority was exclusively detected in the current study (Supplementary Fig. S6).

Since majority of the sampling was carried out in minimal media during exponential and stationary phases of growth, without any subcellular fractionation, our dataset has a bias against sporulation-related or membrane-bound proteins. However, most proteins that were not detected in our study were presumably not present in the analyzed samples under the growth conditions and in the media used. Notably, many of them are uncharacterized (Supplementary Fig. S1); since they are not essential under normal growth conditions, they are likely expressed in response to specific stimuli.

New genes could be formed through duplication-divergence process or via mutations in non-coding DNA sequences^44^, implying that all extant genomes contain a mixture of genes from different evolutionary ages. In this context, genomic phylostratigraphy is an approach that aims to trace the origin of protein families based on similarity searches of a well-populated protein sequence database^45^. It relies on the model of punctuated evolution of protein families which assumes that founder proteins with novel protein sequences regularly emerge in genomes and initiate protein families at different evolutionary levels^44,45^. For example, this method has been successfully used to show that genes of similar evolutionary age also cluster in terms of expression patterns^46^, and that Serine/Threonine protein kinases have a deep evolutionary root^47^. In this study, we observed that core metabolic functions are carried out by proteins that were present in last universal common ancestor and can be found in the oldest phylostrata in Fig. 3. These evolutionary older proteins tend to be expressed in standard laboratory conditions. Genes that are more recent additions to the core genome are populating more recent phylostrata. Proteins encoded by these “younger” genes tend to not be expressed under standard experimental conditions and are probably triggered in specific conditions that led to their inclusion in the genome, i.e. specific stress conditions or environmental challenges.

Protein phosphorylation and acetylation seem to be prominent PTMs in *B. subtilis*. Out of 257 *B. subtilis* proteins encoded by essential genes^48^, 254 are either phosphorylated, acetylated, or modified by both modifications. Notably, phosphorylation of histidine, aspartate or arginine residues was not addressed due to our sample preparation workflow, which was not suitable to analyze such acid-labile forms of phosphorylation. Despite this, phosphorylation on these residues may be present in our dataset in low abundance. GO analysis of the phosphorylation and acetylation events revealed that a significant portion of central metabolic pathways might be regulated by these modifications. Interestingly, several members of the phosphate assimilation pathway (PhoA, PhoB, PhoD, PstS) were detected in phosphorylated form, pointing to a potential regulatory feedback mechanism. Acetylation is known to play a major role in regulating enzymes that form a crucial part of the bacterial metabolism, as seen in the case of *Escherichia coli*^49^, *Salmonella enterica*^19^ or *Mycobacterium tuberculosis*^50^. From an evolutionary perspective (Fig. 3), acetylation and phosphorylation are present at relatively high and constant levels on proteins from the oldest phylostrata, which contain the bulk of housekeeping genes, including those involved in the core metabolism^51,52^. The distribution of these PTMs is much more variable in more recent phylostrata, 6–12, whereas the proteins that are traced back to most recent phylostrata (ps14) were neither phosphorylated nor acetylated. This might indicate that developing recognition of the new proteins by the modifying enzymes (kinases, acetyl-transferases) may take some evolutionary time.

Sequence motifs have the potential to provide important information regarding protein function. Here, 16 potential motif patterns were detected in case of lysine acetylated peptides. An EK(ac)(D/Y/E) motif was recently reported to be observed amongst *B. subtilis* acetylated proteins^24^. Presence of glutamate in the -1 position and tendency of aspartate or glutamate to be in the +1 position was also observed in our dataset. However, there was a higher propensity of leucine or lysine to be present in the +1 position instead. Presence of lysine in the ±3, ±4, ±5 and ±6 positions was also observed. While currently non-enzymatic acetylation is considered to be the prevalent mode of regulation^53,54^, it has also been hypothesized that the internal environment of the bacterial cell helps maintain the positive charge on lysine thus possibly preventing non-enzymatic acetylation via nucleophilic substitution^24^. AcsA in *B. subtilis* has been reported to be multiply modified non-enzymatically as well as in an AcuA-catalyzed reaction^55^. Four of those events were observed in our dataset as well. Comparison of our dataset to acP-dependent acetylation events reported in *E. coli*^54^ resulted in 1% (N = 76) overlap at the site level. Thus, irrespective of the mechanism, acetylation alters protein function and both modes are equally important for understanding the biological properties of a protein. Recently, four new KATs (Nε-lysine acetyltransferases) have been identified in *E. coli*^56^. While RimI in *E. coli* has an ortholog in *B. subtilis*, BLASTp analysis of YiaC, YjaB, and PhnO against *B. subtilis*, resulted in matches with a low alignment score and low identity (<40%). However, AcuA, a known *B. subtilis* acetyltransferase, was found to have 39% identity with YjaB.

The proteome coverage achieved in this study allowed us to perform *B. subtilis* genome re-annotation by proteogenomics, such as done in other bacteria by our group and others^57–61^. As previously reported, target-decoy approach substantially underestimates the FDR in six-frame searches of bacterial genomes^59^. Thus, we required a maximal PEP of 0.0006 for novel PSMs, which corresponded to the median PEP of PSMs from the target database and was substantially lower than the median PEP of PSMs from the six-frame database (median = 0.0047) (Supplementary Fig. S4 and Supplementary Data S2). A number of re-annotated ORFs did not display any ribosome binding sites (RBS) and were not a part of known operons, such trend was also observed for known *B. subtilis* ORFs. Therefore, we hypothesized that the presence of RBS and membership in a known operon are poor predictors in the context of novel ORFs discovery. In addition, putative novel ORFs were significantly shorter (average = 130.7 amino acid length) compared to reference ORFs (average = 293.85). A possible explanation as to why novel ORF are still being identified in a model organism such as *B. subtilis* is that these novel ORFs do not have the same characteristics as the majority of the known ORFs, and therefore present a difficulty for ORF prediction software^58^. It should be noted that the RT-PCR performed in our study did not provide strand information and could be the result of an mRNA transcribed from the opposite strand or even from an operon spanning the genomic region of interest. In this context, the RT-PCR validation was merely used to show the presence of mRNA at the locus corresponding to our selected novel ORFs. Notably, the three pre-filtering ORFs that could not be validated by RT-PCR and sequencing had been identified only due to modified peptides. These validation results emphasize the need for strict filtering, such as maximum PEP threshold and removal of identification only by modified peptide, to filter-out the high number of false positive hits arising from six-frame searches. Among our validated candidate novel ORFs, seq_51322 (genomic location 937,898–938,104 bp) was the most promising because it had the highest number of novel peptides among all uncharacterized ORFs. While we currently cannot provide information on the function of this uncharacterized ORF, we hope this finding to inspire follow-up studies that focus on sporulation phenotype (based on the known product of this operon).

## Methods

Briefly described below are the experimental conditions and data analysis strategies (extended methods can be found in Supplementary Method S1). Notably, this manuscript includes some published datasets [PeptideAtlas ID: PASS00350^36^; ProteomeXchange identifier PXD003764^34^; ProteomeXchange identifier PXD002559^15^; *B. subtilis* SILAC dataset^62^].

### Growth Conditions

Bacterial cells were grown in either of the following growth mediums: (1) chemically defined minimal medium; (2) LB medium (Roth); (3) M9 minimal medium. Stable isotope labeling, of certain samples, was done with isotopically labeled L-lysine (^12^C_6_^14^N_2_ or 4,4,5,6-D4 or ^13^C_6_^15^N_2_). Cells were grown at 37 °C at 200 rpm and harvested by centrifugation at different stages of growth (lag phase, transition phase, logarithmic phase or stationary phase). Growth conditions for each LC-MS/MS run can be found in Supplementary Data S1.

### Protein Extraction And Digestion

Cell lysis was performed either by: (1) resuspension in Y-PER reagent, or (2) resuspension in a SDS lysis buffer. Cell debris were removed by centrifugation. Protein extract was cleaned up by chloroform/methanol precipitation and dissolved in urea and thiourea. Protein concentration was measured by Bradford protein assay. For in-solution digestion, the protein extract was reduced with DTT and alkylated with IAA^37^. Proteins were digested with an endoprotease (Lys-C and/or Trypsin or ArgC for acetylome). Peptides obtained from in-solution digestion were separated into 12 fractions based on their isoelectric point (pI) using the 3100 Offgel Fractionator. Peptides were acidified using acetonitrile (ACN), acetic acid and TFA. Samples for in-gel digestion were separated on a NuPAGE® Bis-Tris 4–12% gradient gel followed by coomassie staining. Cut gel slices were destained and dehydrated with ACN, reduced with DTT and alkylated with IAA. Protein digestion was carried out overnight. Peptides were eluted from the gel using TFA, acetic acid and ACN.

### Phosphopeptide Enrichment

Phosphorylated peptides were enriched for by either of the following methods - (1) titanium dioxide (TiO_2_) chromatography^63^; (2) phospho-tyrosine antibodies^34^; (3) HAMMOC^64^; (4) Prime-XS protocol^65^.

### Acetylated Peptide Enrichment

Digested samples were subjected to solid-phase extraction using Sep-Pak Classic C18 cartridges. Enrichment of acetylated lysine peptides was performed using Acetyl Lysine Agarose Antibody. Agarose beads were incubated with the sample overnight at 4 °C, loaded onto a spin column, washed and peptides were eluted with TFA. C18 discs were activated with methanol and equilibrated with ACN and TFA^66^. The sample was loaded onto the membrane and washed. Peptides were eluted in ACN and acetic acid, concentrated in a vacuum centrifuge and acidified.

### Mass Spectrometric Analysis

Samples were measured on an Easy-LC nano-HPLC coupled to an LTQ-Orbitrap Elite or LTQ-Orbitrap XL mass spectrometer^59,67^. Chromatographic separation was done on a PicoTip fused silica emitter packed with reversed-phase ReproSil-Pur C18-AQ resin. The peptides were injected onto the column at a flow rate of 200 nL/min or 500 nL/min and 280 bars. Peptides were then eluted using a 90 (Elite) or 130 (XL) min segmented gradient. Separated peptides were ionized by electrospray ionization in the positive mode. The mass spectrometer was operated on a data-dependent mode. Survey full-scans for the MS spectra were recorded in the Orbitrap mass analyzer between 300 and 2,000 Thompson at a resolution of 120,000 or 60,000. Top 20 or top 5 most intense peaks were selected for fragmentation with HCD in the HCD cell or with CID in the linear ion trap analyser.

### LC-MS/MS Runs Data Processing

Data processing strategy is outlined in the Supplementary Fig. S7 and consisted of three separate processings. For the first processing (proteome, phosphoproteome and acetylome characterization), acquired MS spectra (1,688 LC-MS/MS runs) were processed with MaxQuant and Andromeda software suite^26,68^. Database search was performed against a target-decoy database of *B. subtilis subtilis* str. 168 obtained from UniProt (4,197 protein entries) and commonly observed laboratory contaminants (245 entries). For the second processing (kinases and phosphatases interaction network), a subset of LC-MS/MS runs (631 files), comprised exclusively of SILAC labelled experiment, was re-processed. For the third processing (proteogenomics analysis), ORFs on all six frames of *Bacillus subtilis* subsp. *subtilis* str. 168 genome were generated and translated. All 1,688 LC-MS/MS runs were re-processed with MaxQuant software against three databases containing *B. subtilis* UniProtKB proteins (4,197 entries; “target” database), six-frame ORFs (254,598 entries; “novel” database) and common lab contaminants (245 entries).

Parameters that were common to all processings are detailed below. Lys-C, Trypsin or ArgC were chosen as endoproteases. When appropriate, three isotopic forms of lysine (Lys0, Lys4, Lys8) were defined as label in group-specific parameters. Oxidation of methionines, N-terminal acetylation, phosphorylation on serine, threonine and tyrosine residues and acetylation on lysine residues were specified as a variable modification (when appropriate). Carbamidomethylation on cysteines was defined as a fixed modification. Re-quantify was enabled (except for the proteogenomics anylsis). A false discovery rate of 1% was applied at the peptide, protein, phosphorylated site and acetylated site levels individually.

### Extraction Of Modification-Specific Sequence Motifs

Motif-x^29,30^ was employed to determine the presence of characteristic motifs within phosphorylated and acetylated peptides. Only localized sites were chosen for the analysis and tested against a background *B. subtilis* subsp. *subtilis* str. 168 database (UniProt).

### Proteogenomics Re-Annotation Workflow

Following proteogenomics database search (see above), peptides were classified as known or novel according to their database of origin (“target” or “novel”). We then integrated protein BLASTP and TBLASTN^69^, Levenshtein distance and neighbouring gene analyses using a dedicated bioinformatics pipeline (see below and Supplementary Method S1). Novel ORFs were explained by (1) amino acid variant, (2) alternative start site, (3) erroneous termination, (4) annotated in other bacteria, or (5) remaining unexplained. Our bioinformatics pipeline is available online^70^.

### Phylostratigraphic Analysis

*Bacillus subtilis* subsp. *subtilis* str. 168 genome (4,177 genes) was mapped onto a consensus phylogeny that spans 15 ps starting from the origin of cellular organisms (ps1) and ending at the origin of *Bacillus subtilis* subsp. *subtilis* group (ps15). The consensus phylogeny was constructed following the phylogenetic literature^71^. Sequence similarity search was performed against a curated and filtered non-redundant (nr) database (NCBI) containing 113,834,351 protein sequences with the BLASTP algorithm at e-value cut-off of 1E−03. Phylogenetically most-distant BLAST match was used as a criterion to assign the stage of evolutionary origin to a gene.

## Electronic supplementary material

Supplementary Information

Supplementary Data S1

Supplementary Data S2

Supplementary Data S3

## Data Availability

The mass spectrometry proteomics data have been deposited to the ProteomeXchange Consortium via the PRIDE^72^ partner repository with the dataset identifier PXD008860. The bioinformatics pipeline, used for proteogenomics re-annotation, is available online^70^.
