# Supplementary material for: In-depth analysis of Bacillus subtilis proteome identifies new ORFs and traces the evolutionary history of modified proteins
Source: Sci Rep. 2018 Nov 22;8:17246. doi: 10.1038/s41598-018-35589-9 (PMC6250715; doi:10.1038/s41598-018-35589-9)

Supplementary information for:

In-depth analysis of *Bacillus subtilis* proteome identifies new ORFs and traces the evolutionary history of modified proteins

*Vaishnavi Ravikumar^1#^, Nicolas C. Nalpas^2#^, Viktoria Anselm^2^, Karsten Krug^2§^, Maša Lenuzzi^3^,* *Martin Sebastijan Šestak^3^*, *Tomislav Domazet-Lošo^3,4^, Ivan Mijakovic^1,5*^, Boris Macek^2*^*

^1)^ Novo Nordisk Foundation Center for Biosustainability, Technical University of Denmark, Kongens Lyngby, Denmark.

^2)^ Proteome Center Tuebingen, Interfaculty Institute for Cell Biology, University of Tuebingen, Germany.

^3)^ Laboratory of Evolutionary Genetics, Ruđer Bošković Institute, Bijenička cesta 54, HR-10000 Zagreb, Croatia.

^4)^ Catholic University of Croatia, Ilica 242, HR-10000 Zagreb, Croatia.

^5)^ Systems and Synthetic Biology, Department of Chemical and Biological Engineering, Chalmers University of Technology, Gothenburg, Sweden.

^§^present address: Proteomics Platform, The Broad Institute of MIT and Harvard, Cambridge MA, USA

#Both authors contributed equally to this work.

*To whom correspondence should be addressed:

Prof. Dr. Boris Macek

Proteome Center Tuebingen

Interfaculty Institute for Cell Biology

Auf der Morgenstelle 15

72076 Tuebingen

Germany

Phone: +49/(0)7071/29-70558

Fax: +49/(0)7071/29-5779

E-Mail: boris.macek@uni-tuebingen.de

Prof. Dr. Ivan Mijakovic

Novo Nordisk Foundation Center for Biosustainability

Technical University of Denmark

Kemitorvet, Building 220

2800 Kongens Lyngby

Denmark

Phone: +46(0)709828446

E-Mail: ivmi@biosustain.dtu.dk

# Supplementary legends

**Supplementary Method S1:** The extended materials and methods used for this study.

**Supplementary Figure S1: Pathway and function distribution of *Bacillus subtilis* proteins.** KEGG pathways were attributed to proteins and distribution was visualized as a pie chart for each of the following groups, namely, a) reference (theoretical) proteome, b) proteins identified in this study, c) proteins undetected in this study, d) identified phosphoproteome, e) identified acetylome; f) The Gene Ontology (GO) cellular component terms were tested for over-representation across the detected (all identified proteins), undetected (proteins never identified in our study), phosphorylated (all proteins found phosphorylated at least once) and acetylated (all proteins found acetylated at least once) proteome categories.

**Supplementary Figure S2: Distribution of identified phosphorylation and acetylation events.** a) Pie chart representing the number of phosphorylation events on all identified proteins. b) Distribution of localized serine, threonine and tyrosine phosphorylation events. c) Pie chart representing the number of acetylation (K) events on all identified proteins. d) Distribution of peptide sequence per number of detected phosphorylation (S/T/Y) events. e) Distribution of peptide sequence per number of detected acetylation (K) events.

**Supplementary Figure S3: Sequence motif analysis for phosphorylated and acetylated peptides.** Over-represented motifs in a +/-6 amino acid sequence window surrounding the localized modified residue (center position) using motif-x. a) Peptides phosphorylated at serine residue. b) Peptides acetylated at lysine residue.

**Supplementary Figure S4: Novel peptides and ORFs classification.** a) Histogram depicting the number of MS/MS scans per nucleotide detected in this study. b) The PEP distribution for the different databases used in the MaxQuant processing. c) The evidence PEP density displayed per database together with the maximum PEP threshold used for quality filtering. d) The number of unique novel peptides and ORFs before and after PEP quality filtering. e) The number of unique novel ORFs before and after PEP quality filtering for each novelty explanation type. f) The potentially novel ORFs are colour coded based on their novelty explanation. In addition, the novel ORFs frequency is displayed per bin of peptide count (higher peptide count results in increase identification certainty).

**Supplementary Figure S5: The genomic region visualization for seq_154909 and seq_49263.** The genomic region visualization for a) seq_154909 and b) seq_49263. Top panel includes known ORFs (in blue for + strand and in red for - strand) and all ORFs generated from six-frame genome translation (in green for + strand and in orange for - strand); color lightness corresponds to whether ORF was identified or not in our data (dark color for expressed ORF and light color for non-expressed ORF). The middle panel is zoomed around the expressed novel ORF of interest for visualization of peptide sequences (in khaki are peptides mapping to novel ORF and in purple are peptides from known ORF) and RT-PCR nucleotide sequences at this genomic location. The bottom panel contains the MS/MS spectra of the top scoring novel PSM.

**Supplementary Figure S6: Comparison to other published datasets.** Venn diagram showing overlap of the current study with published a) proteomes, b) transcriptome, c) phosphoproteome, and d) acetylomes of *Bacillus subtilis*. Interestingly, there were four proteins (L8E9J0 - Biofilm-forming protein, L8EAY3 - Uncharacterized protein, L8ECQ9 - Regulator of iron homeostasis, Q9K3A9 - Uncharacterized membrane protein YdzM/YouB) identified in the current study that have not been previously confirmed to be expressed.

**Supplementary Figure S7: LC-MS/MS data processing workflow.** Three separate data processings were performed in this study to perform: 1) proteome, phosphoproteome, acetylome and phylostratigraphy analyses (blue workflow); 2) kinases and phosphatases interaction network analysis (orange workflow); 3) proteogenomics reannotation analysis (green workflow).

**Supplementary Data S1 (XLSX)**: List of all proteins, phosphorylation events and acetylation events identified in this study. A list of all LC-MS/MS runs is also provided together with their respective growth conditions.

**Supplementary Data S2 (XLSX): Lists for the proteogenomics analyses performed in this study.** Sheet 1: The complete novel evidence information list. The MaxQuant evidence table for the entries mapped exclusively to six-frame database to which information from the current proteogenomics pipeline were included, such as the database of origin for specific evidences and the novelty explanation. Sheet 2: The identified novel ORFs table. All identified novel ORFs are listed together with their peptide count, minimum evidence PEP, maximum evidence score, intensity, nucleotide and amino acid length, peptide and ORF novelty explanation, along with relevant clues. Sheet 3: the details of primers used for RT-PCR.

**Supplementary Data S3 (ZIP): Sanger sequencing results for the validated novel ORFs.**

Sanger sequencing results of the RT-PCR samples that successfully amplified. Results contain the following novel ORFs: a) seq_49263, b) seq_51322, c) seq_145510 and d) seq_154909. Each visualization contains on the first track the novel ORF genomic sequence as well as location of the primers used for RT-PCR reactions. The subsequent tracks represent the sequence of RT-PCR amplicons (including base quality).

# Supplementary Method S1

This manuscript partly includes some published datasets (PeptideAtlas ID: PASS00350 ^1^; ProteomeXchange identifier PXD003764 ^2^; ProteomeXchange identifier PXD002559 ^3^; *Bacillus subtilis* SILAC dataset ^4^). Strains used, sampling and extraction conditions, sample preparation details and instrument settings are described in respective papers for each of the above mentioned datasets. Described below are experimental conditions for the rest of the data (also see **Supplementary Data S1**).

## Growth Conditions

Bacterial cells were grown in either of the following growth media -
(1) chemically defined minimal medium containing 15 mM ammonium sulphate, 2 mM calcium chloride, 1 μM ferrous sulphate, 8 mM magnesium sulphate, 10 μM manganese sulphate, 27 mM potassium chloride, 0.6 mM potassium dihydrogen phosphate, 7 mM sodium citrate (Merck), 50 mM Tris-HCl pH 7.5 (Sigma Aldrich) supplemented with 0.5 % glucose (AppliChem), 0.67 mM glutamic acid (Merck) and 490 μM tryptophan (Sigma Aldrich);
(2) Luria-Bertani (LB) medium (Roth);

(3) synthetic M9 (Sigma Aldrich) minimal medium supplemented with 0.5 % glucose, 1 mM magnesium sulphate, 0.1 mM calcium chloride and 1 % thiamine (Sigma Aldrich).

Stable isotope labeling, of certain samples, was done by supplementing the minimal medium with 0.025 % of the respective isotopically labeled L-lysine (^12^C_6_ ^14^N_2_ or 4,4,5,6-D4 or ^13^C_6_ ^15^N_2_) (Euriso-Top or SILANTES). Cells were grown at 37 °C at 200 rpm and harvested at different stages of growth (lag phase, transition phase, logarithmic phase or stationary phase). *B. subtilis* 168 strains used in this manuscript include Δ*lysA*; Δ*lysA* Δ*yveL*; Δ*lysA* Δ*yfkJ*; Δ*lysA* Δ*yabT*; Δ*lysA* Δ*ybdM*. Cells were harvested by centrifugation at 7000 x *g* for 10 min.

## Protein Extraction

Cell lysis was performed by either of the two methods stated below:
(1) Cell pellets were resuspended in Y-PER (Thermo Fisher Scientific) reagent supplemented with 50 μg/mL of lysozyme, 5 mM of phosphatase inhibitors - sodium fluoride (SF) and glycerol-2-phosphate (GP) and protease inhibitor cocktail. Cells were lysed by incubation at 37 °C for 20 min.
(2) Cell pellets were resuspended in an SDS lysis buffer containing 4 % SDS in 100 mM triethylammonium bicarbonate (TEAB) pH 8.6, 5 mM GP, 5 mM SF, 5 mM sodium orthovanadate (SOV), 10 mM ethylenediaminetetraacetic acid (EDTA) and protease inhibitor cocktail. The cell extract was then boiled at 90 °C for 10 min.

Cell lysis was followed by sonication for 30 s at 40 % amplitude on ice. The cell debris was removed by centrifugation at 13.4 rpm for 30 min. The crude protein extract was cleaned up by chloroform/methanol precipitation and the proteins were dissolved in denaturation buffer containing 6 M urea and 2 M thiourea in 10 mM Tris-HCl pH 8.0. Protein concentration was measured by Bradford protein assay (Bio-Rad Laboratories).

## Protein Digestion

Protein digestion was performed either in-gel or in-solution. In-solution digestion was carried out as described previously ^5^. Briefly, the protein extract was reduced with 1 mM dithiothreitol (DTT) and alkylated with 5.5 mM iodoacetamide (IAA) in the dark, for 1 h each at room temperature. Proteins were then predigested with an endoprotease (Lys-C (Wako GmbH) and/or Trypsin (Promega) for proteome and phosphoproteome analysis or ArgC (Roche) for acetylome analysis) for 3 h followed by overnight digestion at room temperature with the same.

Peptides obtained from in-solution digestion were separated based on differences in their isoelectric point (pI) using the 3100 Offgel Fractionator (Agilent). Peptides were separated into 12 fractions using 13 cm Immobiline Drystrips (pH gradient 3-10) (GE Healthcare) using the default settings with a maximum current of 50 μA and potential difference of 20 kVh. Fractionated peptides were acidified using 30 % acetonitrile (ACN), 5 % acetic acid and 10 % trifluoroacetic acid (TFA).

Samples for in-gel digestion were separated on a NuPAGE® Bis-Tris 4-12 % gradient gel (Invitrogen) following the manufacturer’s instructions. Gel electrophoresis was carried out at 200V constant for 1 h followed by coomassie staining. Cut gel slices were destained with 5 mM ammonium bicarbonate (ABC) in 50 % ACN (1:1, v/v) and dehydrated with 100 % ACN followed by reduction with 10 mM DTT in 20 mM ABC and alkylation with 55 mM IAA in 20 mM ABC. Protein digestion was carried out overnight. Peptides were eluted from the gel using 3 % TFA in 30 % ACN, 0.5 % acetic acid in 80 % ACN and 100 % ACN.

## Phosphopeptide Enrichment

Phosphorylated peptides were enriched for by - (1) titanium dioxide (TiO_2_) chromatography with 2,5-dihydroxybenzoic acid (DHB) ^6^ or 6 % TFA ^7^; (2) phospho-tyrosine antibodies ^2^; (3) HAMMOC ^8^; (4) Prime-XS (http://www.primexs.eu/downloads/Public-Documents/04---Protocols/PRIME-XS-Protocol-TiO2-phosphopeptide-enrichment/). Briefly, TiO_2_ spheres of 10 μm were pre-incubated with an appropriate competitive binder, such as DHB, 6 % TFA or lactic acid on an end-over-end rotation wheel followed by incubation with the sample. Incubated beads were then washed with appropriate buffers and the phosphorylated peptides were eluted from the beads under basic pH conditions. Additionally, enrichment specifically for phosphorylated tyrosines was carried out using the PTMScan® Phospho-Tyrosine antibody according to the manufacturer’s instructions.

## Acetylated Peptide Enrichment

Digested samples were first subjected to solid-phase extraction using Sep-Pak Classic C18 cartridges (Waters). Enrichment of acetylated lysine peptides was performed using Acetyl Lysine Agarose Antibody (ImmuneChem). Samples were diluted in a buffer containing 500 mM MOPS pH 7.2, 100 mM sodium phosphate and 500 mM sodium chloride. Pre-equilibrated agarose beads were incubated with the diluted sample overnight at 4 °C. Incubated beads were next loaded onto a spin column (Sigma Aldrich) and washed multiple times with buffer and water. Acetylated peptides were subsequently eluted from the beads with 0.15 % TFA.

All samples were stage-tipped ^9^ prior to loading them onto the mass spectrometer. Briefly, C18 discs (Empore^TM^) were activated with methanol and equilibrated with 2 % ACN, 1 % TFA. The sample was loaded onto the membrane and washed with 0.5 % acetic acid. Peptides were eluted in 80 % ACN, 0.5 % acetic acid, concentrated in a vacuum centrifuge, acidified and subjected to nano-LC-MS/MS measurements.

## Mass Spectrometric Analysis

Samples were measured on an Easy-LC nano-HPLC (Proxeon Biosystems) coupled to an LTQ-Orbitrap Elite or LTQ-Orbitrap XL mass spectrometer (Thermo Fisher Scientific), as described previously ^10,11^. Chromatographic separation was done on a 15 cm PicoTip fused silica emitter with an inner diameter of 75 μm and a tip diameter of 8 μm, packed in-house with reversed-phase ReproSil-Pur C18-AQ 3 μm resin (Dr. Maisch GmbH). A column oven was attached to the column to maintain a steady temperature of the column at 30 °C. The peptides were injected onto the column with 0.5 % acetic acid at a flow rate of 200 nL/min for the LTQ-Orbitrap Elite and 500 nL/min for the LTQ-Orbitrap XL, at 280 bars. Peptides were then eluted using a 90 (LTQ-Orbitrap Elite) or 130 (LTQ-Orbitrap EXL) min segmented gradient (5 – 90 %) of 80 % ACN, 0.5 % acetic acid, the flow rate being maintained constant at 200 nL/min. Separated peptides were ionized by electrospray ionization or ESI in the positive mode. Ion spray voltage was set at 2.35 kV. Capillary voltage and temperature were set to 2.3 kV and 275 °C. The mass spectrometer was operated on a data-dependent mode. Survey full-scans for the MS spectra were recorded in the Orbitrap mass analyzer between 300 and 2,000 Thompson at a resolution of 120,000 or 60,000 with a target value of 10^6^ charges. The top 20 or top 5 most intense peaks from the survey scans were selected for fragmentation with higher-energy collisional dissociation (HCD) in the HCD cell using nitrogen gas or with collision induced dissociation (CID) in the linear ion trap analyzer using helium gas, with a collision energy of 35 %. A target value of 5,000 charges was set for each scan cycle. A 60 s dynamic exclusion was set on the LTQ-Orbitrap Elite and 90 s on the LTQ-Orbitrap XL. For phosphoproteomics analysis, ions were fragmented by multi stage activation or MSA with neutral loss occurring at −97.97, −48.98 and −32.66. Additionally, lock masses option was enabled on the LTQ-Orbitrap XL for internal calibration ^12^ and to improve the mass accuracy.

## LC-MS/MS Runs Data Processing

All raw data analyzed in this study have been generated in the laboratory of Boris Macek, Proteome Center Tübingen, University of Tübingen, Germany. These were used for three independent processing that are outlined in the **Supplementary Fig. S7**.

Firstly, a total of 1,688 MS runs were processed. Acquired MS spectra were processed with the MaxQuant software suite (version 1.5.1.0) ^13^, integrated with the Andromeda search engine ^14^. Database search was performed against a target-decoy database of *B. subtilis* subsp. *subtilis* str. 168 obtained from UniProt (taxonomy ID 224308), containing 4,197 *B. subtilis* protein entries and 245 commonly observed laboratory contaminants. Lys-C, Trypsin or ArgC were chosen as the endoproteases, with a maximum missed cleavage of two specified. Three isotopic forms of lysine (light, medium and heavy) were stated in the search space. Oxidation of methionines, N-terminal acetylation, phosphorylation on serine, threonine and tyrosine residues and acetylation on lysine residues were specified as a variable modification. Initial maximum allowed mass tolerance was set to 12 ppm for the precursor and 0.5 Da for the fragment ions. Carbamidomethylation on cysteines was defined as a fixed modification. Re-quantify was enabled. A false discovery rate of 1 % was applied at the peptide, protein, phosphorylated site level and acetylated site level individually and only fragments with a minimum length of seven amino acids were used for SILAC peptide quantification. A minimum of two unmodified peptide counts was set as a requirement for the respective protein quantification.

Secondly, a subset of the entire dataset was re-processed and consisted of 613 LC-MS/MS runs from SILAC studies. All MaxQuant parameters described above remained identical for this re-processing. The results of this processing were used to construct a network of kinases, phosphatases and their respective substrates.

Thirdly, to carry out proteogenomics analysis, we downloaded the genome of *Bacillus subtilis* subsp. *subtilis* str. 168 from the European Nucleotide Archive (reference: GCA_000009045.1). Open Reading Frames (ORFs) on all six frames were generated and translated using getorf tool (translation table XI) from the Emboss software package ^15^. In addition, we defined a minimum threshold length of seven amino acids for compatibility with subsequent MS database search. All previously described LC-MS/MS runs (1,688 runs) were then processed with the MaxQuant software ^16^ via the Andromeda search engine ^14^ against three databases containing all UniProtKB proteins for *B. subtilis* subsp. *subtilis* str. 168 (4,197 entries), all putative ORFs (254,598 entries) and common lab contaminants (245 entries), respectively. MaxQuant parameters were set identically as detailed above.

## Protein over-representation analysis

The different proteome datasets (detected, undetected, phosphorylated and acetylated) were tested for over-representation against the KEGG pathways and gene ontologies (GO) ^17,18^. The protein annotation were obtained using the UniProt.ws package ^19^. The over-representation was done via the clusterProfiler package ^20^ based on hypergeometric distribution (Benjamini-Hochberg *p*-adj. ≤ 0.05). We performed the analysis in the R environment ^21^.

## Motif extraction

Motif-x ^22,23^ was employed to extract sequence motifs within the phosphorylated and acetylated peptides. Only localized sites were chosen for the analysis. A sequence window containing 6 amino acids upstream and downstream of the phosphorylated or acetylated residue was chosen as data input. For serine, threonine and tyrosine phosphorylated peptides, default parameters of an occurrence threshold of 20 and p-value threshold (for the binomial probability) of 1.00E−06 were maintained. For lysine acetylated peptides, p-value threshold was set to 1.00E−08 and occurrences to 200. The sequences were tested against a background *B. subtilis* subsp. *subtilis* str. 168 database (taxonomy ID 224308) obtained from UniProt. Peptides phosphorylated on serine, threonine and tyrosine residues and acetylated on lysine residues were analyzed individually by defining the central character as S, T, Y or K respectively.

## Proteogenomics re-annotation workflow

After proteogenomics database search (see above), we classified all peptides according to their databases of origin using the following rules: (1) remaining peptides matching to UniProtKB proteins were classified as “known”; (2) remaining peptides matching to contaminant proteins were classified as “contaminant”; (3) remaining peptides matching to putative ORFs were classified as “novel”; and (4) peptides matching to reverse database were classified as “reverse”. Each peptide position within respective proteins was determined using the stringr R package. To limit the number of false positive present in our identified potentially novel peptides, we established a maximum posterior error probability (PEP) threshold based on the median PEP of known peptides. In addition, all protein groups and associated peptides that were identified only due to modified peptides were filtered out.

Subsequently, we performed and integrated a number of analyses in order to explain each potentially novel peptide. Firstly, we aligned all putative ORFs to protein entries in the known database using blastp tool ^24,25^. Secondly, we calculated the Levenshtein distance of the potentially novel peptides against protein entries in the known database. Thirdly, we aligned all identified putative ORFs to all UniProtKB and RefSeq protein sequences using blastp. The results of these analyses allowed stratification into peptides containing single amino acid variant (SAV), peptides matching an alternative start site region, peptides corresponding to erroneous termination (or alternative termination site), peptides annotated in other bacterial species or strains and peptides that remain unexplained.

We then prioritized putative ORFs by counting the number of peptide hits; the principle being that putative ORFs with higher number of peptide hits are more likely to be true positive. Putative ORFs with more than one peptide hits were subsequently manually validated, meaning that the proteogenomics workflow explanation for these entries was confirmed through literature and web resources curation. The putative ORFs that showed discrepancies in explanation between the proteogenomics workflow and manual annotation or remained unexplained were selected for further experimental validation by RT-PCR.

Exact genomic coordinates for the known proteins and six-frame ORFs were calculated from alignment of all known proteins and six-frame ORFs to *B. subtilis* subsp. *subtilis* str. 168 genome using tblastn tool. Additional analysis made use of BSgenome ^26^ and GenomicRanges ^27^ packages to compute overall genome and per nucleotide coverage. This also allowed for finding overlap between six-frame ORFs and operon genomic position—operon information were obtained from the database of prokaryotic operons ^28^. The motifRG package ^29^ was used to identify over-represented motifs between −30 and +2 bp of known genes; which should correspond to Ribosome Binding Sites (RBS). The six-frame ORFs were searched for presence of RBS motifs in vicinity of potential start codon.

Finally the ggbio package ^30^ was used for circos and linear track representation of known proteins and putative ORFs on the genome. The complete bioinformatics pipeline used for this analysis was performed in Bash shell and R environment ^21^, and is available online ^31^.

## Validation of putative novel ORFs

*B. subtilis* subsp. *subtilis* str. 168 was inoculated in 5 mL LB media and cultured at 37 °C and 200 rpm overnight. Cells were diluted by a factor of 1000 in LB media (17 µL overnight culture into 17 mL LB media). OD_600_ was then measured after 2-3 h and approximately 4 × 10^8^ cells were harvested (collect 666.7 µL at OD_600_ = 0.6). Cells were lysed enzymatically and total RNA was harvested using RNAprotect Bacteria Reagent and RNeasy Mini Kit (Qiagen) according to the manufacturer’s instructions. RNA quantity and quality were assessed, revealing a 260/280 ratio greater than 2.0 and concentration of 124 ng/µL. RNA sample was stored at −80 °C until needed. cDNA was prepared from 800 ng extracted RNA using QuantiTect Reverse Transcription Kit (Qiagen), including genomic DNA elimination, according to the manufacturer’s instructions.

PCR was performed using Taq DNA polymerase with ThermoPol buffer (NEB) on a T100™ Thermal cycler (Bio-Rad) according to manufacturer’s instructions. Reactions contained 4 μL diluted cDNA sample (approximately 5 ng/µL), 1X of ThermoPol buffer, 1 µL of 10 mM dNTPs, 200 nM final concentration of each primer (**Supplementary Data S2**), 1.25 units of Taq polymerase and nuclease free water for a total volume of 50 µL. PCR cycling conditions comprised (1) a 95 °C step for 30 s, (2) 34 cycles at 95 °C for 30 s, 50 °C for 30 s and 68 °C for 1 min, and (3) a 68 °C step for 5 min. PCR reactions were purified using mi-PCR Purification Kit (Metabion). RT-PCR products sequence was confirmed by Sanger sequencing (GATC Biotech).

## References

1 Ravikumar, V. *et al.* Quantitative phosphoproteome analysis of Bacillus subtilis reveals novel substrates of the kinase PrkC and phosphatase PrpC. *Mol Cell Proteomics* **13**, 1965-1978, doi:10.1074/mcp.M113.035949 (2014).

2 Shi, L., Ravikumar, V., Derouiche, A., Macek, B. & Mijakovic, I. Tyrosine 601 of Bacillus subtilis DnaK Undergoes Phosphorylation and Is Crucial for Chaperone Activity and Heat Shock Survival. *Front Microbiol* **7**, 533, doi:10.3389/fmicb.2016.00533 (2016).

3 Rosenberg, A. *et al.* Phosphoproteome dynamics mediate revival of bacterial spores. *BMC Biol* **13**, 76, doi:10.1186/s12915-015-0184-7 (2015).

4 Soufi, B. *et al.* Stable isotope labeling by amino acids in cell culture (SILAC) applied to quantitative proteomics of Bacillus subtilis. *J Proteome Res* **9**, 3638-3646, doi:10.1021/pr100150w (2010).

5 Macek, B. *et al.* The serine/threonine/tyrosine phosphoproteome of the model bacterium Bacillus subtilis. *Mol Cell Proteomics* **6**, 697-707, doi:10.1074/mcp.M600464-MCP200 (2007).

6 Macek, B. *et al.* Phosphoproteome analysis of E. coli reveals evolutionary conservation of bacterial Ser/Thr/Tyr phosphorylation. *Mol Cell Proteomics* **7**, 299-307, doi:10.1074/mcp.M700311-MCP200 (2008).

7 Sharma, K. *et al.* Ultradeep human phosphoproteome reveals a distinct regulatory nature of Tyr and Ser/Thr-based signaling. *Cell Rep* **8**, 1583-1594, doi:10.1016/j.celrep.2014.07.036 (2014).

8 Nakagami, H. StageTip-based HAMMOC, an efficient and inexpensive phosphopeptide enrichment method for plant shotgun phosphoproteomics. *Methods Mol Biol* **1072**, 595-607, doi:10.1007/978-1-62703-631-3_40 (2014).

9 Ishihama, Y., Rappsilber, J. & Mann, M. Modular stop and go extraction tips with stacked disks for parallel and multidimensional Peptide fractionation in proteomics. *J Proteome Res* **5**, 988-994, doi:10.1021/pr050385q (2006).

10 Franz-Wachtel, M. *et al.* Global detection of protein kinase D-dependent phosphorylation events in nocodazole-treated human cells. *Mol Cell Proteomics* **11**, 160-170, doi:10.1074/mcp.M111.016014 (2012).

11 Krug, K. *et al.* Deep coverage of the Escherichia coli proteome enables the assessment of false discovery rates in simple proteogenomic experiments. *Mol Cell Proteomics* **12**, 3420-3430, doi:10.1074/mcp.M113.029165 (2013).

12 Olsen, J. V. *et al.* Parts per million mass accuracy on an Orbitrap mass spectrometer via lock mass injection into a C-trap. *Mol Cell Proteomics* **4**, 2010-2021, doi:10.1074/mcp.T500030-MCP200 (2005).

13 Cox, J. *et al.* A practical guide to the MaxQuant computational platform for SILAC-based quantitative proteomics. *Nat Protoc* **4**, 698-705, doi:10.1038/nprot.2009.36 (2009).

14 Cox, J. *et al.* Andromeda: a peptide search engine integrated into the MaxQuant environment. *J Proteome Res* **10**, 1794-1805, doi:10.1021/pr101065j (2011).

15 Rice, P., Longden, I. & Bleasby, A. EMBOSS: the European Molecular Biology Open Software Suite. *Trends Genet* **16**, 276-277 (2000).

16 Cox, J. & Mann, M. MaxQuant enables high peptide identification rates, individualized p.p.b.-range mass accuracies and proteome-wide protein quantification. *Nat Biotechnol* **26**, 1367-1372, doi:10.1038/nbt.1511 (2008).

17 Kanehisa, M., Sato, Y., Kawashima, M., Furumichi, M. & Tanabe, M. KEGG as a reference resource for gene and protein annotation. *Nucleic Acids Res* **44**, D457-462, doi:10.1093/nar/gkv1070 (2016).

18 Kanehisa, M., Furumichi, M., Tanabe, M., Sato, Y. & Morishima, K. KEGG: new perspectives on genomes, pathways, diseases and drugs. *Nucleic Acids Res* **45**, D353-D361, doi:10.1093/nar/gkw1092 (2017).

19 UniProt.ws: R Interface to UniProt Web Services v. R package version 2.18.0 (2017).

20 Yu, G., Wang, L. G., Han, Y. & He, Q. Y. clusterProfiler: an R package for comparing biological themes among gene clusters. *OMICS* **16**, 284-287, doi:10.1089/omi.2011.0118 (2012).

21 R: A language and environment for statistical computing (R Foundation for Statistical Computing. <http://www.R-project.org>, Vienna, Austria, 2013).

22 Schwartz, D. & Gygi, S. P. An iterative statistical approach to the identification of protein phosphorylation motifs from large-scale data sets. *Nat Biotechnol* **23**, 1391-1398, doi:10.1038/nbt1146 (2005).

23 Chou, M. F. & Schwartz, D. Biological sequence motif discovery using motif-x. *Curr Protoc Bioinformatics* **Chapter 13**, Unit 13 15-24, doi:10.1002/0471250953.bi1315s35 (2011).

24 Altschul, S. F., Gish, W., Miller, W., Myers, E. W. & Lipman, D. J. Basic local alignment search tool. *Journal of molecular biology* **215**, 403-410, doi:10.1016/S0022-2836(05)80360-2 (1990).

25 Camacho, C. *et al.* BLAST+: architecture and applications. *BMC Bioinformatics* **10**, 421, doi:10.1186/1471-2105-10-421 (2009).

26 BSgenome: Infrastructure for Biostrings-based genome data packages and support for efficient SNP representation v. R package version 1.44.0 (2017).

27 Lawrence, M. *et al.* Software for computing and annotating genomic ranges. *PLoS Comput Biol* **9**, e1003118, doi:10.1371/journal.pcbi.1003118 (2013).

28 Mao, F., Dam, P., Chou, J., Olman, V. & Xu, Y. DOOR: a database for prokaryotic operons. *Nucleic Acids Res* **37**, D459-463, doi:10.1093/nar/gkn757 (2009).

29 motifRG: A package for discriminative motif discovery, designed for high throughput sequencing dataset v. R package version 1.22.0 (2012).

30 Yin, T., Cook, D. & Lawrence, M. ggbio: an R package for extending the grammar of graphics for genomic data. *Genome Biol* **13**, R77, doi:10.1186/gb-2012-13-8-r77 (2012).

31 Nalpas, N. & Macek, B. A complete proteogenomics pipeline for bacterial genome re-annotation. doi:10.5281/zenodo.1312851 (2018).

# Supplementary Figure S1


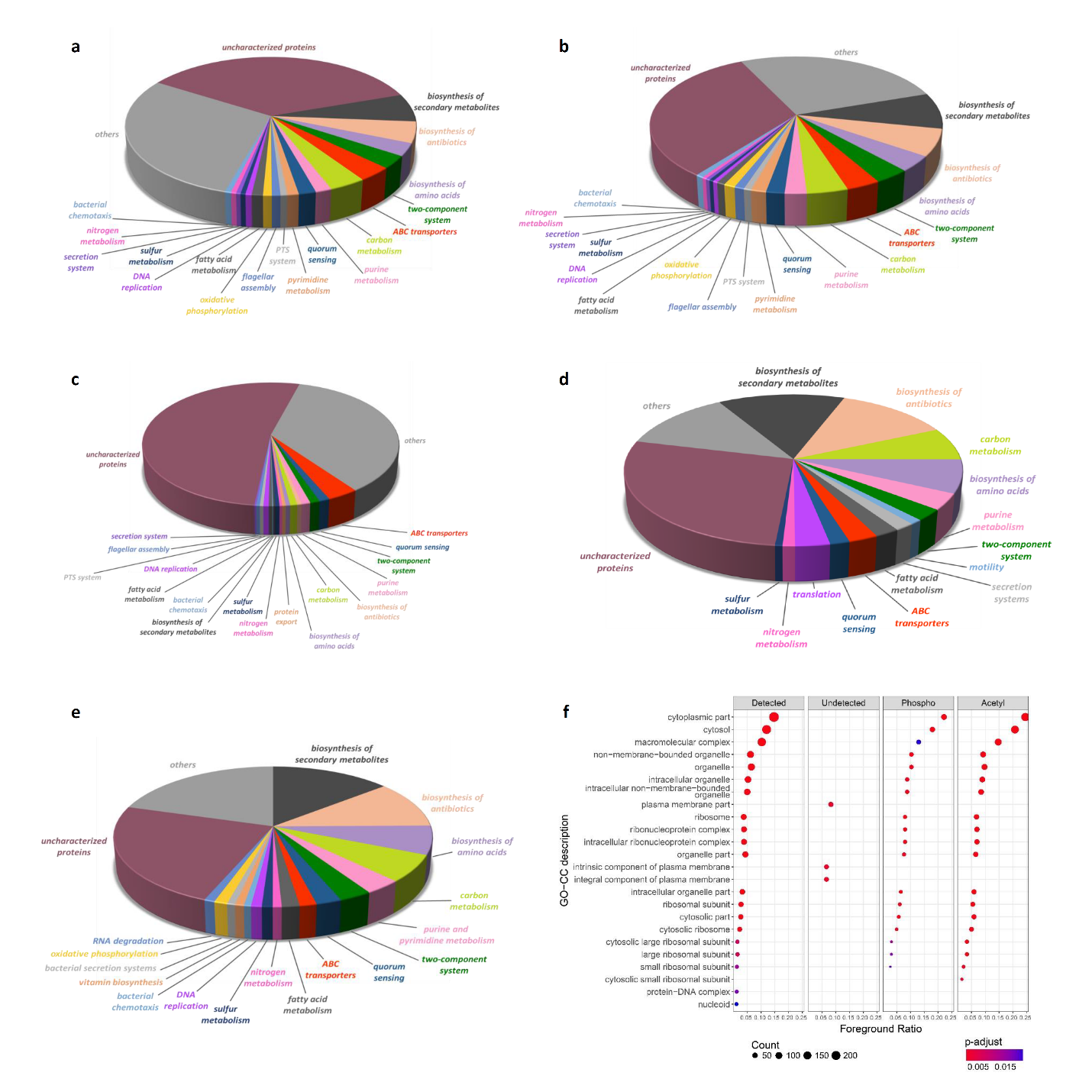


# Supplementary Figure S2


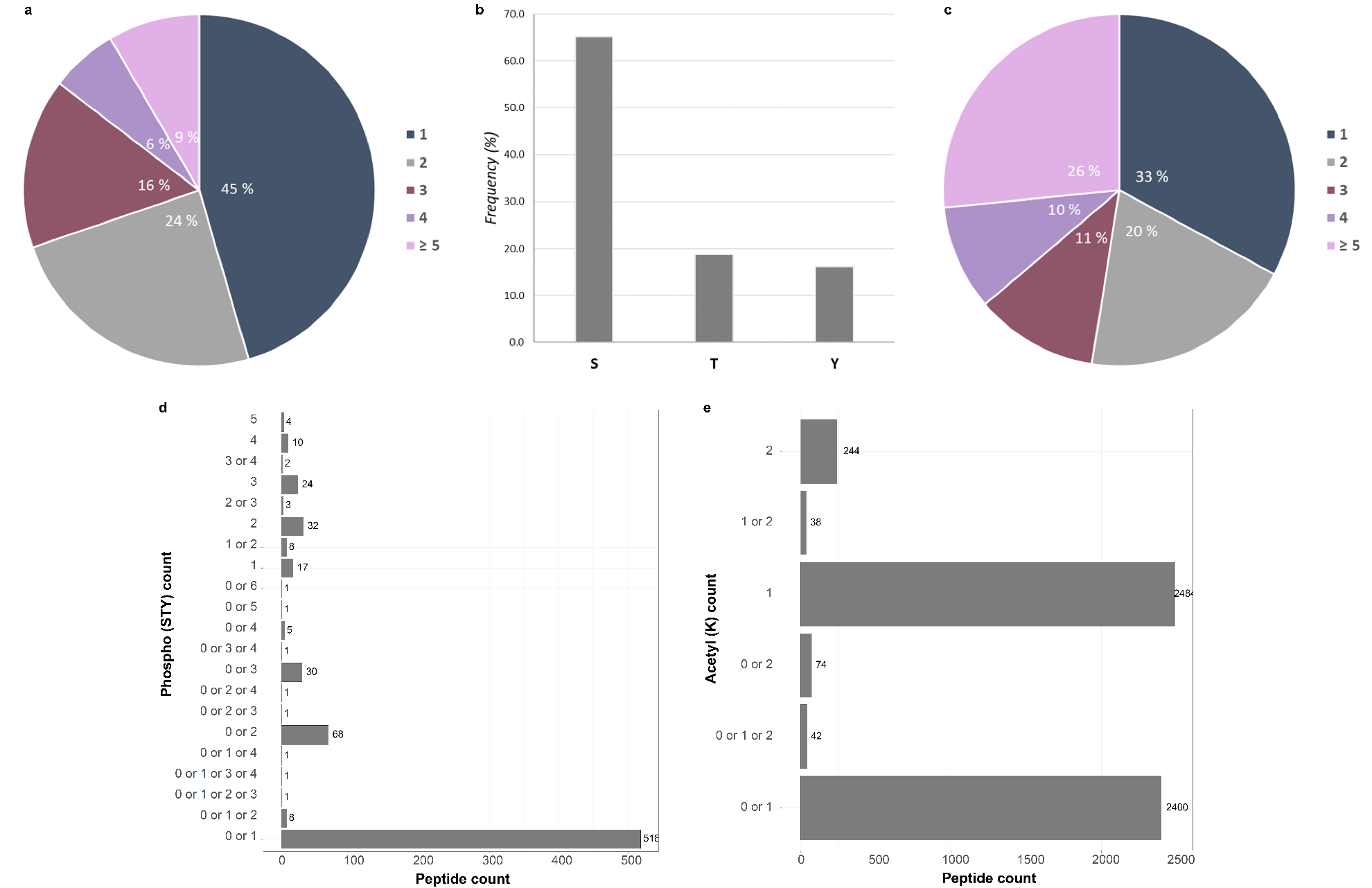


# Supplementary Figure S3


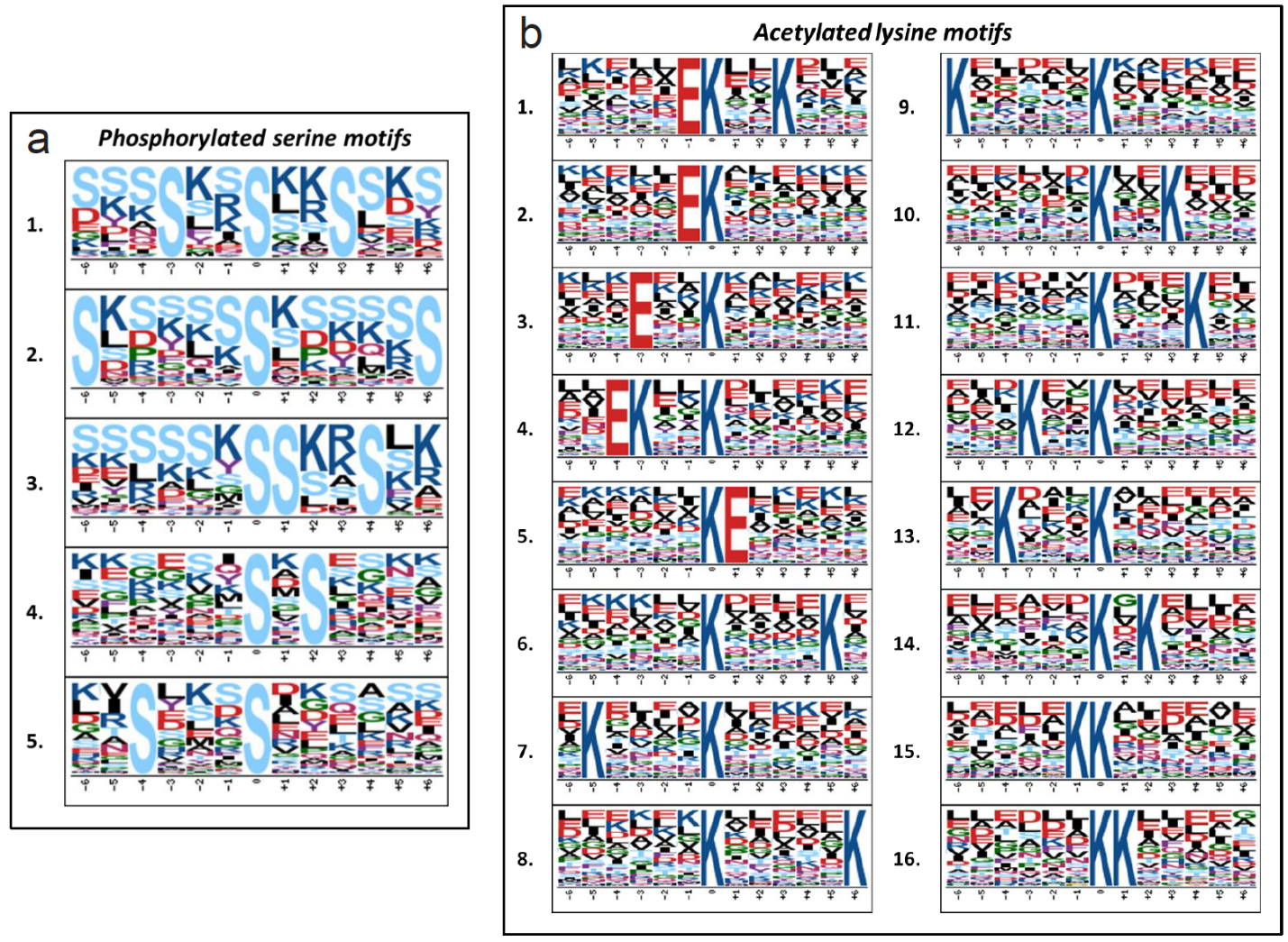


# Supplementary Figure S4


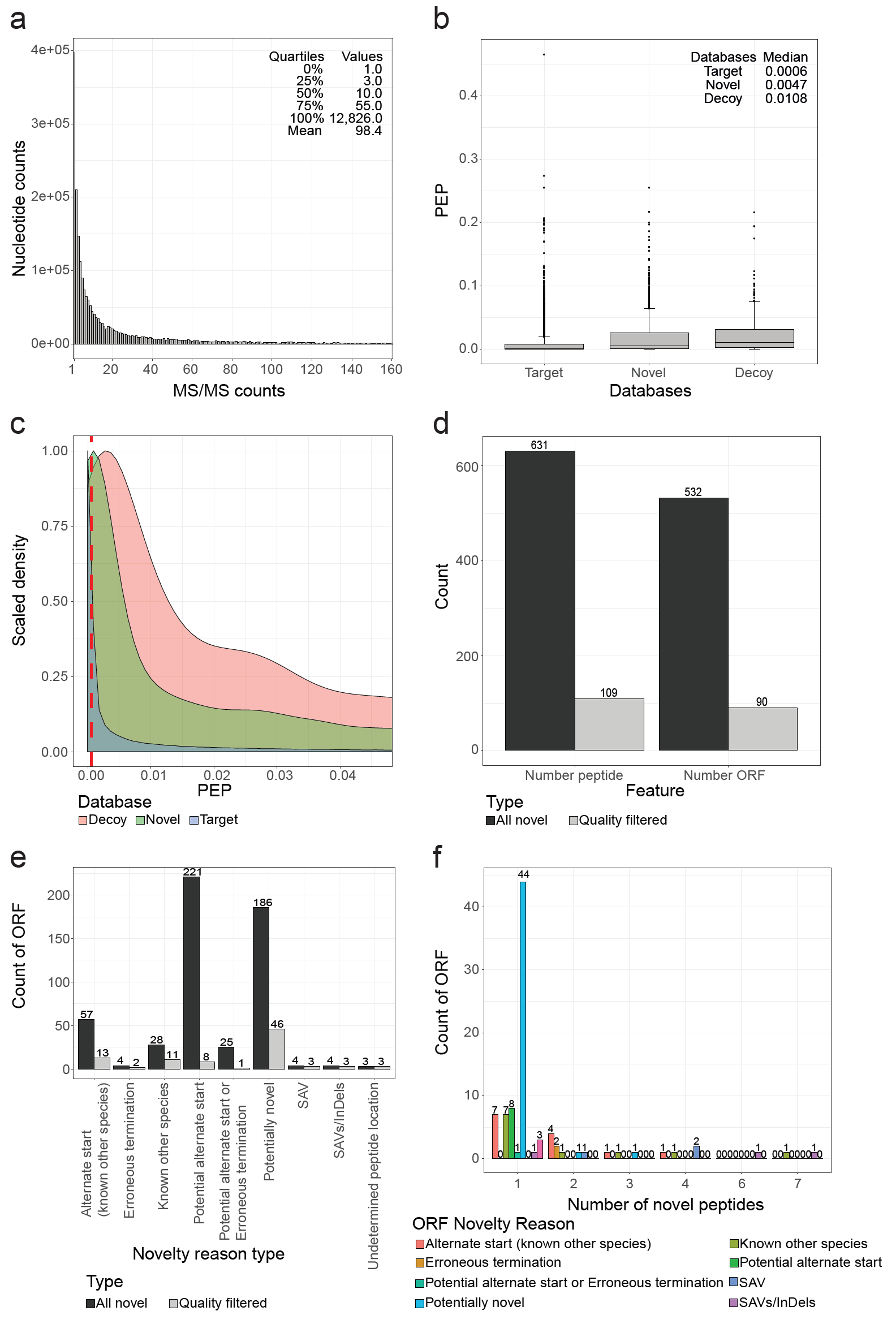


# Supplementary Figure S5


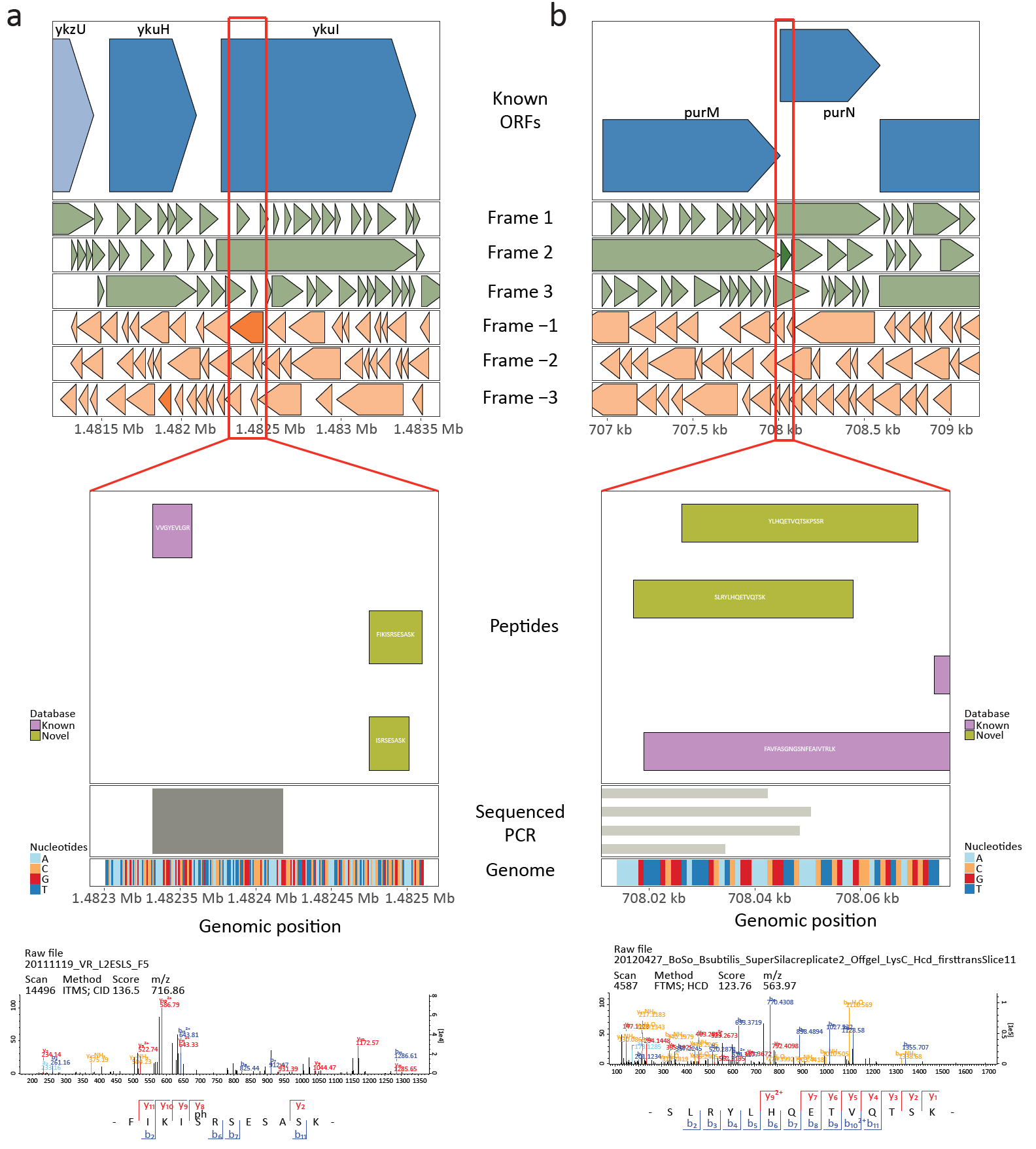


# Supplementary Figure S6


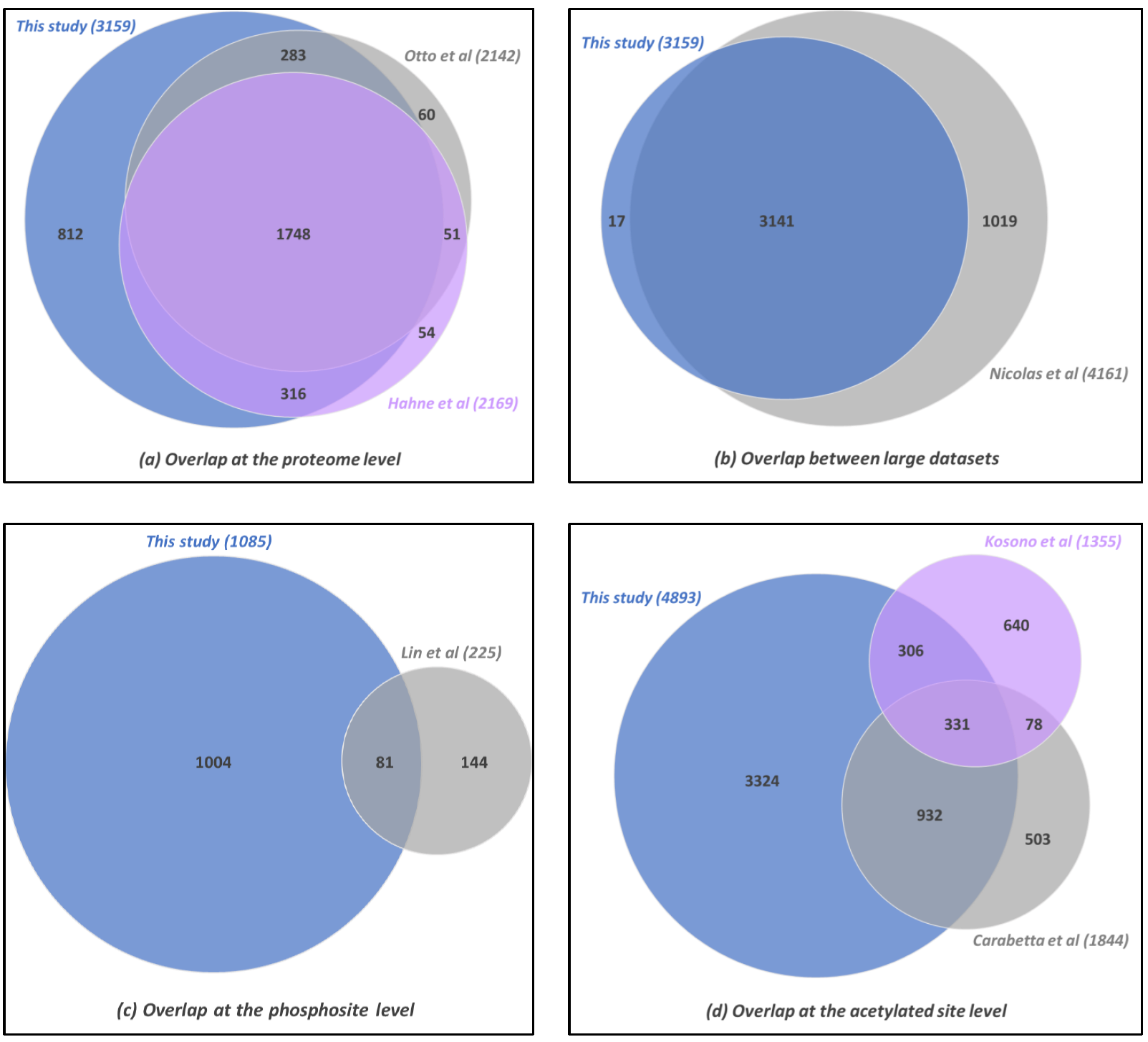


# Supplementary Figure S7


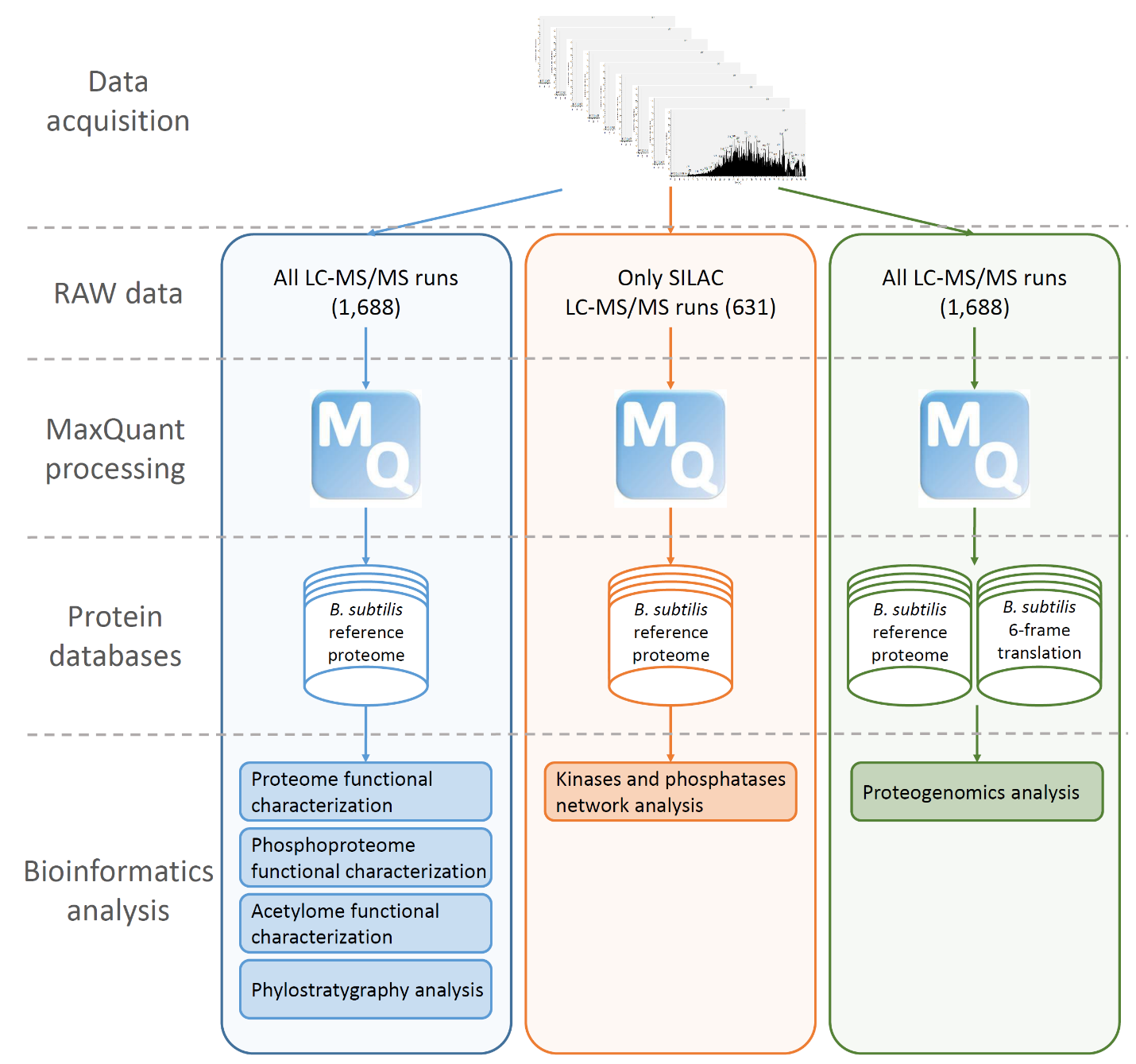

Supplement: Supplementary file 1 — Supplementary Information [file 41598_2018_35589_MOESM1_ESM.docx]
